# A Novel Osteochondrodysplasia With Empty Sella Associates With a *TBX2* Variant

**DOI:** 10.3389/fendo.2022.845889

**Published:** 2022-03-03

**Authors:** Riikka E. Mäkitie, Sanna Toiviainen-Salo, Ilkka Kaitila, Outi Mäkitie

**Affiliations:** ^1^ Folkhälsan Institute of Genetics, Helsinki, Finland; ^2^ Research Program for Clinical and Molecular Metabolism, Faculty of Medicine, University of Helsinki, Helsinki, Finland; ^3^ Department of Otorhinolaryngology–Head and Neck Surgery, Helsinki University Hospital and University of Helsinki, Helsinki, Finland; ^4^ Children’s Hospital, Pediatric Research Center, University of Helsinki and Helsinki University Hospital, Helsinki, Finland; ^5^ Medical Imaging Center, Pediatric Radiology, University of Helsinki and Helsinki University Hospital, Helsinki, Finland; ^6^ Department of Medical Genetics, University of Helsinki, Helsinki, Finland; ^7^ Department of Clinical Genetics, Helsinki University Hospital, Helsinki, Finland; ^8^ Department of Molecular Medicine and Surgery and Center for Molecular Medicine, Karolinska Institutet, Stockholm, Sweden; ^9^ Department of Clinical Genetics, Karolinska University Hospital, Stockholm, Sweden

**Keywords:** osteochondrodysplasia, empty sella, TBX2, vertebral abnormalities, osteoarthritis

## Abstract

Skeletal dysplasias comprise a heterogenous group of developmental disorders of skeletal and cartilaginous tissues. Several different forms have been described and the full spectrum of their clinical manifestations and underlying genetic causes are still incompletely understood. We report a three-generation Finnish family with an unusual, autosomal dominant form of osteochondrodysplasia and an empty sella. Affected individuals (age range 24–44 years) exhibit unusual codfish-shaped vertebrae, severe early-onset and debilitating osteoarthritis and an empty sella without endocrine abnormalities. Clinical characteristics also include mild dysmorphic features, reduced sitting height ratio, and obesity. Whole-exome sequencing excluded known skeletal dysplasias and identified a novel heterozygous missense mutation c.899C>T (p.Thr300Met) in *TBX2*, confirmed by Sanger sequencing. TBX2 is important for development of the skeleton and the brain and three prior reports have described variations in *TBX2* in patients portraying a complex phenotype with vertebral anomalies, craniofacial dysmorphism and endocrine dysfunctions. Our mutation lies near a previously reported disease-causing variant and is predicted pathogenic with deleterious effects on protein function. Our findings expand the current spectrum of skeletal dysplasias, support the association of *TBX2* mutations with skeletal dysplasia and suggest a role for TBX2 in development of the spinal and craniofacial structures and the pituitary gland.

## Introduction

Skeletal dysplasias encompass a heterogenous group of developmental disorders of skeletal and cartilaginous tissues. Several different forms have been described but the full spectrum of their clinical manifestations and underlying genetic causes remain elusive. The current classification of the skeletal dysplasias (1) includes 461 different entities but novel skeletal dysplasias, affecting solely the skeletal structures or associated with extra-skeletal involvement, are continuously described.

Three earlier studies have reported genetic variants in the *TBX2* gene to associate with syndromic disorders involving the skeleton. Liu et al. reported two heterozygous *TBX2* missense variants to result in a complex phenotype with craniofacial dysmorphism, vertebral abnormalities and endocrine dysfunction (2). Two other studies reported microdeletions or copy number variants encompassing *TBX2* in patients with skeletal and limb malformations and cardiac defects (3, 4). Genome-wide association studies (GWAS) have further identified SNPs in *TBX2* to associate with height and sitting-height to height ratio (5, 6).

The family of T-box (TBX) genes encodes transcription factors with wide expression in various tissues and essential roles throughout development (7). Multiple genetic syndromes have been shown to associate with defective TBX function, such as *TBX1* in DiGeorge/velocardiofacial syndrome, *TBX3* in ulnar-mammary syndrome, *TBX4* in ischiopatellar dysplasia and *TBX5* in Holt-Oram syndrome (8, 9). TBX2 is especially crucial for embryogenesis and morphogenesis with notable roles in the development of skeletal and craniofacial structures, the brain and cardiac tissues (10). *In vitro* and *in vivo* studies have implicated TBX2 in limb and palate patterning and *Tbx2* shows strong expression in all facial primordia and limb buds (11, 12). Furthermore, *Tbx2* mutant mice exhibit a cleft palate phenotype and malformed digits, before dying prematurely due to cardiac malformations (12, 13). Despite these, and the reported clinical cases, *TBX2*-related skeletal pathology remains poorly characterized and the molecular mechanisms, dialogue between *TBX2* and other pathways and its role in human disorders remain unclear.

This study describes a three-generation Finnish family with an unusual and novel form of skeletal dysplasia with bone and joint involvement and pituitary gland abnormalities. Exome-wide genetic studies revealed a novel missense variant p.Thr300Met in *TBX2* that has not been previously linked to a human disease.

## Subjects and Methods

### Subjects

**Figure 1:**
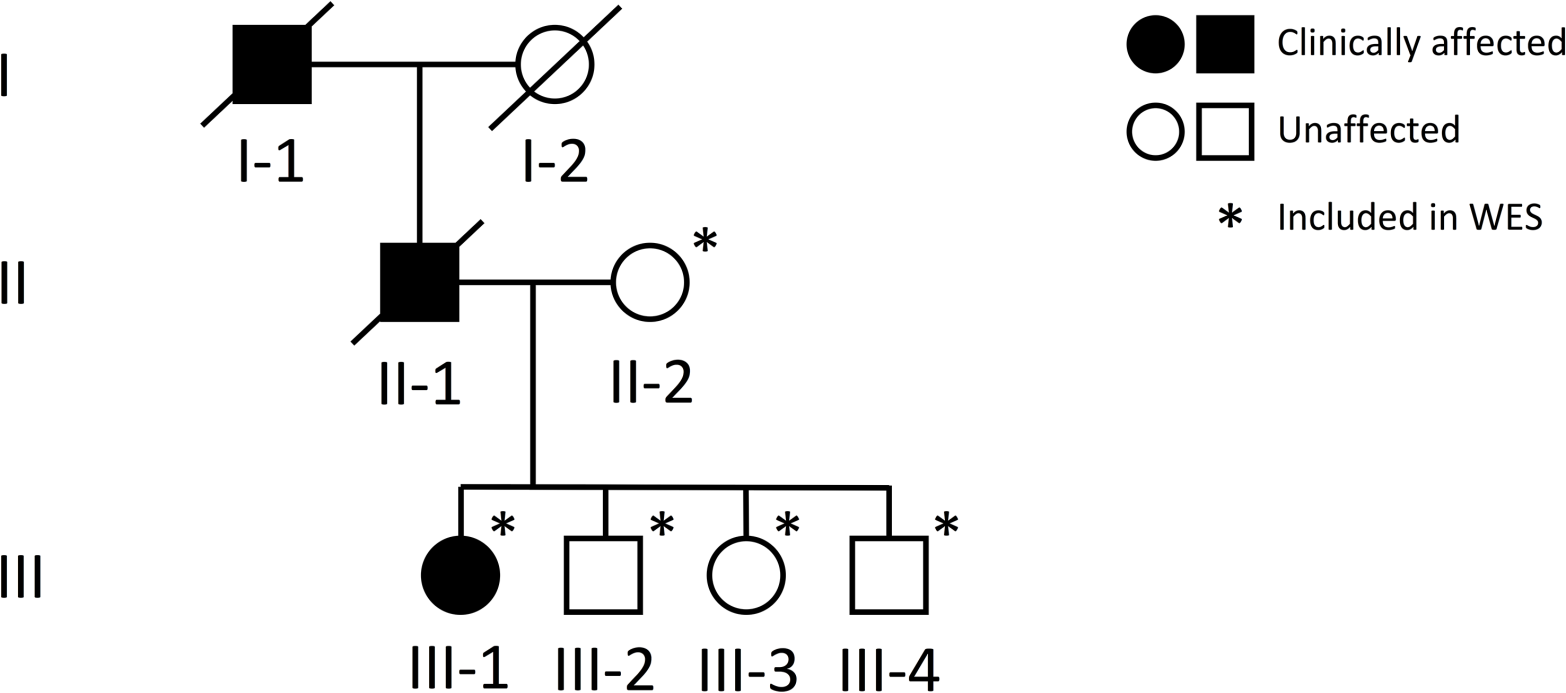
Pedigree of a Finnish family with a novel *TBX2* missense variant p.Thr300Met. Squares represent males, circles females, black symbols clinically affected family members, white symbols clinically unaffected family members, and slashes deceased family members. Generations are numbered with roman numerals. Individuals included in WES analysis are indicated with an asterisk.

The study involves a three-generation Finnish family initially evaluated at Helsinki University Hospital for a suspected autosomal dominant skeletal dysplasia (**Figure 1**). Prior to study enrolment, the participating individuals signed a written informed consent. All clinical and genetic studies were approved by the Institutional Ethics Board at Children’s Hospital, Helsinki University Hospital, Finland.

### Clinical Data

The subjects were clinically evaluated at the Helsinki University Hospital, Finland. Medical histories were collected by patient interview and retrospectively from medical records. Clinical data included skeletal and non-skeletal morbidities, growth and development, past surgeries and long-term medications, and prior imaging studies.

For the index patient, biochemical parameters were first assessed at age 12. After diagnosis of an empty sella, additional hormonal parameters were evaluated at age 13. All parameters were evaluated from peripheral blood and urine in the morning between 8 and 9 a.m., after an overnight fast and using second morning void urine. Biochemistry was measured at HUSLAB laboratories, Helsinki, Finland, and included complete blood count, creatinine, concentrations of calcium and phosphate, parathyroid hormone (PTH), 25-Hydroxyvitamin D (S-25-OH-D; assessed by chemiluminescent immunoassay (CLIA), Abbott, Deerfield, IL, USA), and bone markers including serum total alkaline phosphatase (ALP), serum N-terminal propeptide of type I procollagen (PINP, marker of bone formation; CLIA, IDS-iSYS) and urinary N-telopeptide of type I collagen (U-NTx, marker of bone resorption; enzyme-linked immunosorbent assay ELISA, Abbott). Hormonal parameters included serum concentrations of follicle stimulating hormone (FSH), luteinizing hormone (LH), thyroid stimulating hormone (TSH), thyroxin (T4), estradiol (E2), prolactin (PRL), cortisol, and insulin-like growth hormone factor-1 (IGF-1) and insulin-like growth factor binding protein-3 (IGFBP3) for assessment of possible growth hormone deficiency. Urinary concentrations of calcium were also measured. Biochemistry data for the two other patients (II-1 and I-1) were collected from past medical records.

For the index patient, bone mineral density (BMD) was evaluated with dual-energy X-ray absorptiometry (DXA) for lumbar spine (L1–L4), total hip and total body (Hologic Inc., Bedford, MA, USA). Measured values were converted to Z-scores. Plain skeletal radiographs were obtained for thoracic and lumbar spine and long bones. Vertebral morphology was analyzed from spinal radiographs. Brain structures were evaluated with MR-imaging, as well as the spine and intraspinal structures. For the two other patients, previously obtained radiological evaluations were reviewed.

### Genetic Studies

DNA samples were obtained of the index patient (III-1), her healthy mother (II-2) and her three healthy siblings (III-2, -3, -4). Unfortunately, no DNA was available from the father (II-1) or the grandfather (I-1) who had deceased prior to our study. Genomic DNA was extracted from peripheral blood using standard methods. To search for the genetic cause of the family’s disorder, we proceeded to whole-exome sequencing (WES), for which we included the index, her healthy mother and healthy siblings (**Figure 1**). WES was performed at Blueprint Genetics (Espoo, Finland) according to their standard protocol (blueprintgenetics.com; **Supplementary Material**). The procedure yielded x178 median coverage of target bases. Variant annotation was performed using Variant Effect Predictor (version 84) (14) and variant exploration using the GEMINI framework (0.19.0) (15).

Prior to exome-wide exploration for causative variants, we first screened the data for variants in the 23 genes known to underlie osteogenesis imperfecta (OI), bone fragility and primary osteoporosis (16, 17). We then filtered variants in all genes and selected candidate variants according to the following criteria: 1) heterozygous in the index patient (III-1) and absent in the other family members (II-2, III-3, III-4, III-5); functional variants affecting coding regions or splice junctions; and 3) with an allele frequency of <0.1% in Genome Aggregation Database (gnomAD; v2 and v3) (www.gnomad.broadinstitute.org), the 1000 Genomes Project (18), The Single Nucleotide Polymorphism Database 153 (dsSNP153) (https://ftp.ncbi.nlm.nih.gov/snp/), and the Sequencing Initiative Suomi (SISu) database (www.sisuproject.fi). *In silico* predictions for the damaging capacity for missense variants were performed using SIFT (www.sift.jcvi.org), PROVEAN (http://provean.jcvi.org/index.php), PolyPhen2 (www.genetics.nwh.harvard.edu/pph2/), UMD-Predictor (www.umd.predictor.eu), MutationTaster2), M-CAP (www.http://bejenaro.stanford.edu/mcap/), and CADD scores (www.cadd.gs.washington.edu). The variants’ possible effects on protein conformation were evaluated using VarMap (19) and HOPE web server (http://www.cmbi.ru.nl/hope/). Segregation and trueness of the selected candidate variant was confirmed by Sanger sequencing. Primers and protocols are available upon request from the authors.

Copy number variation (CNV) was analyzed from WES data in all known disease genes also by Blueprint Genetics. These genes are supplemented with genes included in The Clinical Genomics Database (>3350 genes, https://research.nhgri.nih.gov/CGD/) and the Developmental Disorders Genotype-Phenotype Database (DD2GP) (>1640 genes, https://www.sanger.ac.uk/collaboration/deciphering-developmental-disorders-ddd/). The total number of genes considered as clinically associated in the CNV analysis is at least 3750.

## Results

### Clinical Features

**Figure 2:**
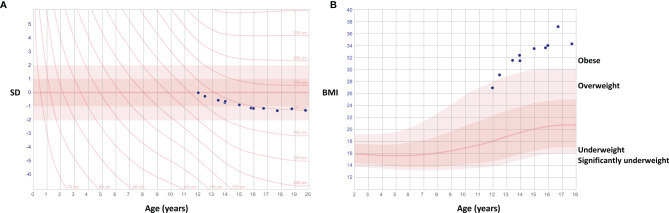
Growth charts of the female patient with a novel *TBX2* missense variant p.Thr300Met. Charts show deceleration of growth after age 12 years with declining height **(A)** and simultaneous weight gain **(B)**.

**The index patient (III-1)**, presently a 24-year-old female, was first evaluated at the age of 12 years for an unusual skeletal dysplasia. She was born full-term, after an uneventful pregnancy and with normal measurements of 3570 g and 51 cm. Although early growth and pubertal development had proceeded normally, at first evaluation at age 12 her stature showed disproportionate body dimensions with a short and straightened back and relatively long limbs, a protruding sternum and narrow shoulders. After age 10 years her growth had started to decelerate, height declining from +1.0 SD to +0.4 SD, with simultaneous weight gain up to 69.6 kg (height-adjusted weight +58%) by age 12 years and 89 kg (90%) by 16 years (**Figure 2**). She had slightly hypotonic musculature, which made her walking stiff and slow. This was further complicated by debilitating pain in multiple joints, which has persisted since early childhood and required continuous pain medication and cortisone injections. She has undergone multiple surgeries including bilateral hip arthroplasty at age 21 and left ankle arthrodesis at age 23. She has also suffered several iritis-type episodes and one episcleritis. She has not been diagnosed with peripheral or vertebral compression fractures. Facial features include slight hypertelorism, depressed nasal bridge and bulbous nasal tip, mild epicanthal folds, low-set and slightly cupped ears, and a narrow palate. Skull shape and size are normal, and no frontal prominence is observed. She has had multiple orthodontic procedures but does not have dentinogenesis imperfecta. She has no cardiac problems. These clinical observations lead to a clinical diagnosis of osteochondrodysplasia and osteoarthritis.

Biochemistry for complete blood count has been repeatedly normal, as has been measurements for electrolytes, calcium and phosphate excluding hypercalciuria (U-Ca/U-Crea 1.22, reference 0.04–0.7) and low vitamin D (25-OHD 25 nmol/L, reference >50 nmol/L), which later normalized with supplementation (**Table 1**). Creatinine has remained low 1–49 µmol/L, likely due to small muscle mass. Biomarkers for rheumatoid arthritis, including HLA-B27, rheumatoid factor and antinuclear antibodies, and for bone turnover are normal. Other hormone concentrations have been normal.

**Table 1:** Biochemistry in three Finnish patients with a novel *TBX2* missense mutation p.Thr300Met.

Patient	III-1	II-1	I-1
**Sex, age**	Female, 24 years	Male, deceased 40 years	Male, deceased 44 years
**Age at evaluation**	13	20	40
**General parameters**			
**Hb [g/L]**	137	163	167
**Leuk [E9/L]**	6.7	7	7.1
**P-Ca-Ion [mmol/L]**	1.27	1.45	Normal
**P-Pi [mmol/L]**	1.55	0.89	N/A
**P-Mg [mmol/L]**	0.92	N/A	N/A
**P-PTH [ng/L]**	26	N/A	N/A
**25-OH-D [nmol/L]**	25	N/A	N/A
**Bone markers**			
**P-ALP [U/L]**	236	157	N/A
**S-PINP [µg/L]**	428	N/A	N/A
**S-ICTP [µg/L]**	15.4	N/A	N/A
**U-NTx [nmol/mmol]**	283	N/A	N/A
**U-Ca/U-Crea [mmol/mmol]**	**1.22**	N/A	N/A
**Hormonal markers**			
**S-FSH [IU/L]**	5.2	Normal	N/A
**S-LH [IU/L]**	6.3	Normal	N/A
**S-TSH [mU/L]**	1.52	Normal	Normal
**S-T4 [pmol/L]**	14	Normal	Mild thyrotoxicosis
**S-E2 [nmol/L]**	0.177	Normal testosterone	N/A
**S-PRL [mU/L]**	331	244	N/A
**P-Cortisol [nmol/L]**	Normal	Normal	N/A
**S-IGF-1 [nmol/L]**	61	Normal	N/A
**S-IGFBP-3 [mg/L]**	5.9	N/A	N/A

Supranormal values are in **bold**, subnormal values are underlined. S, serum; P, plasma. For index patient, reference ranges according to HUSLAB laboratories: Hb (hemoglobin) 116–154, Leuc (lecuocytes) 4.5–13.5; Ca-Ion (calcium ion) 1.16–1.3; Pi (phosphate); Mg (magnesium) 0.7–1; PTH (parathyroid hormone) 8–73; 25-OH-D (25-hydroxyvitamin D) >40; ALP (alkaline phosphatase) 115–435; S-PINP (intact procollagen I N-terminal propeptide; bone formation marker) 400–800; S-ICTP (serum carboxyterminal type I collagen telopeptide) 6–20; U-NTx (urine type I collagen cross-linked N-telopeptide; bone resorption marker) 307–1763; FSH (follicle stimulating hormone) 02–8; LH (luteinizing hormone) 0.5–9; TSH (thyroid stimulating hormone) 0.4–5; T4 (thyroxin) 10–19; EstdioL (estradiol in children) 0.06–0.31; PRL (prolactin) 102–496; IGF-1 (insulin-like growth factor 1) 19–110; IGFBP-3 (Insulin-like growth factor-binding protein 3) 2.9–8.6. NA, Not available.

**Figure 3:**
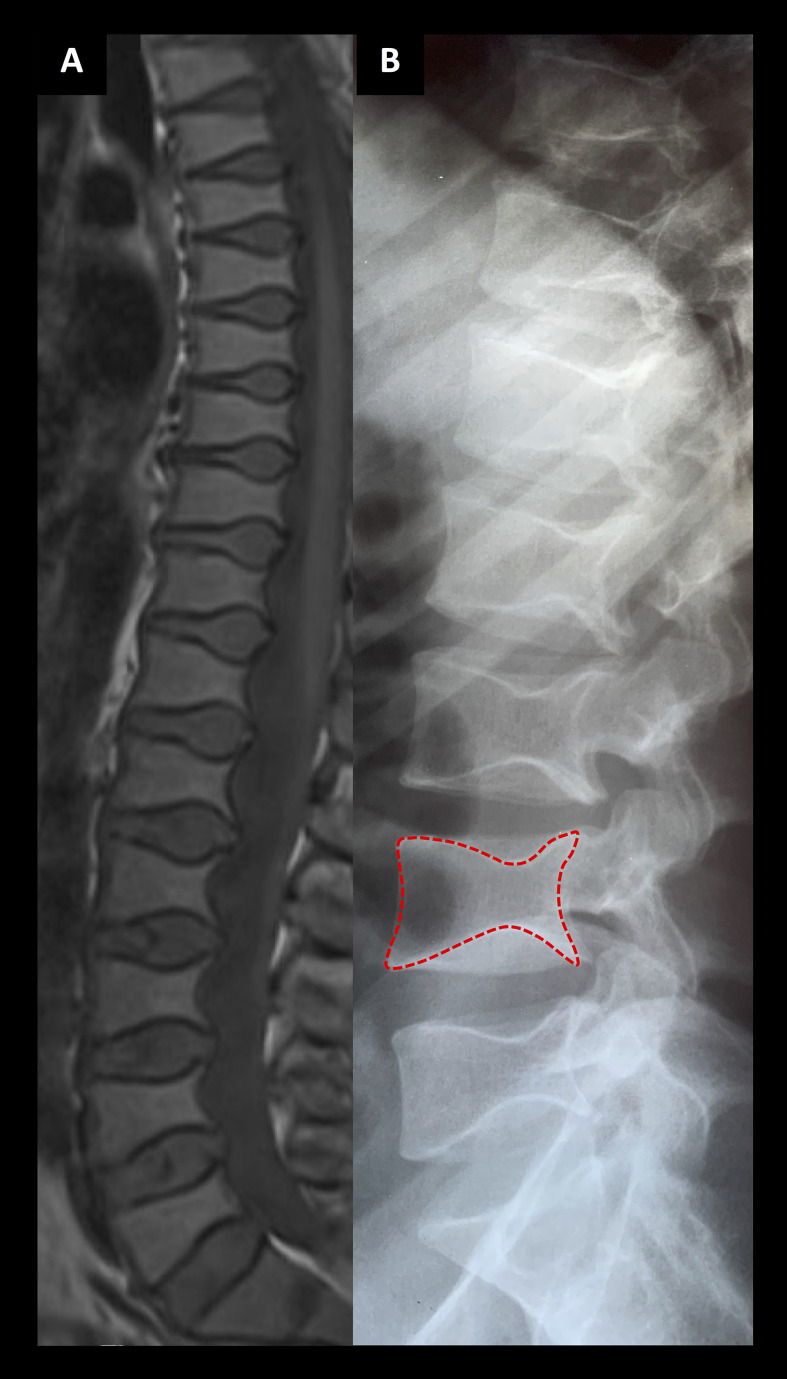
Spinal images of two patients associated with a novel *TBX2* missense variant p.Thr300Met. **(A)** An MRI section of the female index at age 24 years and **(B)** a lumbar radiograph of her father at age 24 years showing codfish-type vertebral dysplasia with balloon-shaped intervertebral discs.

**Figure 4:**
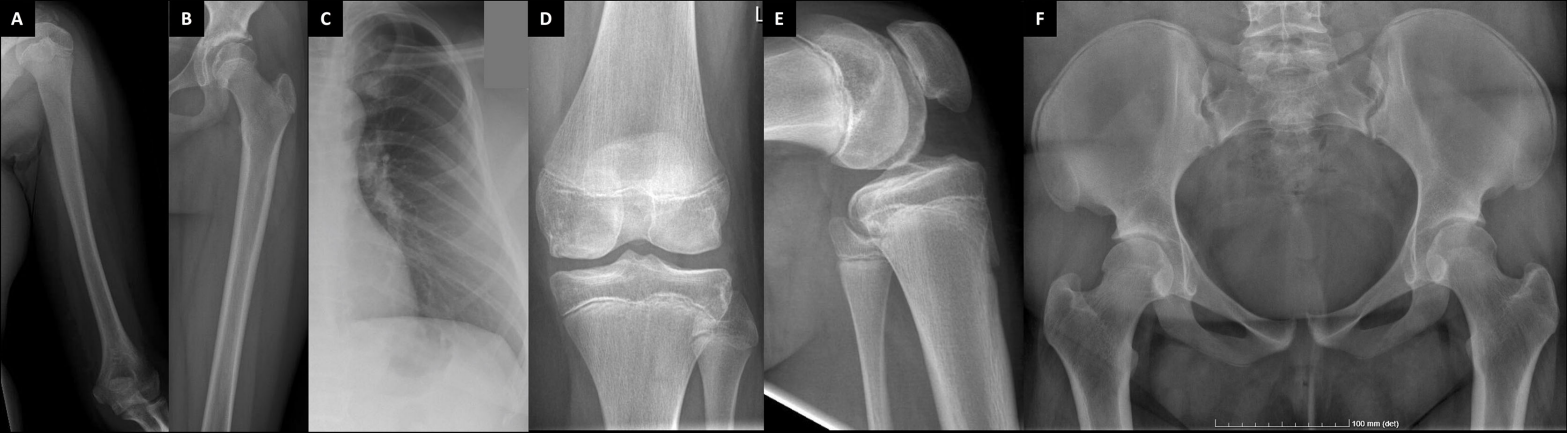
Plain radiographs of the female patient with a novel *TBX2* missense variant p.Thr300Met. The index patient (III-1) at age 11 **(A–C)** and age 24 **(D–F).** Images show slender diaphyses in long-bones and ribs, abnormal shaping in pelvic bones and degenerative changes on articular surfaces in hip and knee joints.

Her skeletal radiographs portray acetabular and epiphyseal irregularities and severely destructed articular surfaces at the hip and knees and wide iliac wings (**Figures 3**, **4**). Spinal images reveal codfish-type skeletal deformity in all thoracic and lumbar vertebrae alongside balloon-shaped intervertebral discs. Long bones and ribs appear slightly long and slender but without visible deformities or deficient mineralization. DXA measurements have been repeatedly normal: Z-scores at age 15, and bisphosphonate-naïve, were +0.4 for lumbar spine, -0.3 for femoral head and +0.3 for whole body.

Furthermore, she has suffered recurrent migraine-like headaches with visual field defects. Brain MRI scans revealed a slightly enlarged and abnormally shaped pituitary fossa filled mostly with cerebrospinal fluid (**Figures 6A**–**C**). Hypophyseal tissue, although with a normal homogenous signal, appears as a thin layer compressed against the sellar floor, representative of a so-called empty sella appearance. Slightly tortuous optic nerves, perioptic nerve sheaths distended with increased amount of cerebrospinal fluid (CSF), and ocular globe flattening (**Figure 6B**), along with the finding of an empty sella, are imaging features that raise the suspicion of idiopathic intracranial hypertension. Although the intracranial pressure was not measured in the index patient, the finding of an empty sella—as well as the headaches and vision defects—could be partly accounted for by increased CSF pressure. No other abnormalities have been noted in cranial structures or the brain tissue.

**The index’s father (II-1)**, deceased at age 40 due to a car accident, had a similar phenotype with skeletal dysplasia, severe early-onset joint degeneration and an empty sella without evident endocrinological abnormalities. He had first undergone bilateral femoral valgus osteotomy and hip arthroplasty at age 23, and later bilateral hip replacement at age 38. He was of normal height, but his back was relatively short (sitting height 47.7%), he had mild thoracic kyphosis with a straightened lumbar lordosis, and his extremities appeared long. He was also overweight (BMI 33.7). No apparent dysmorphic features were mentioned. He had suffered recurrent episodes of headache, but he had no other neurological symptoms, and his cognitive skills and vision were normal.

**Figure 5:**
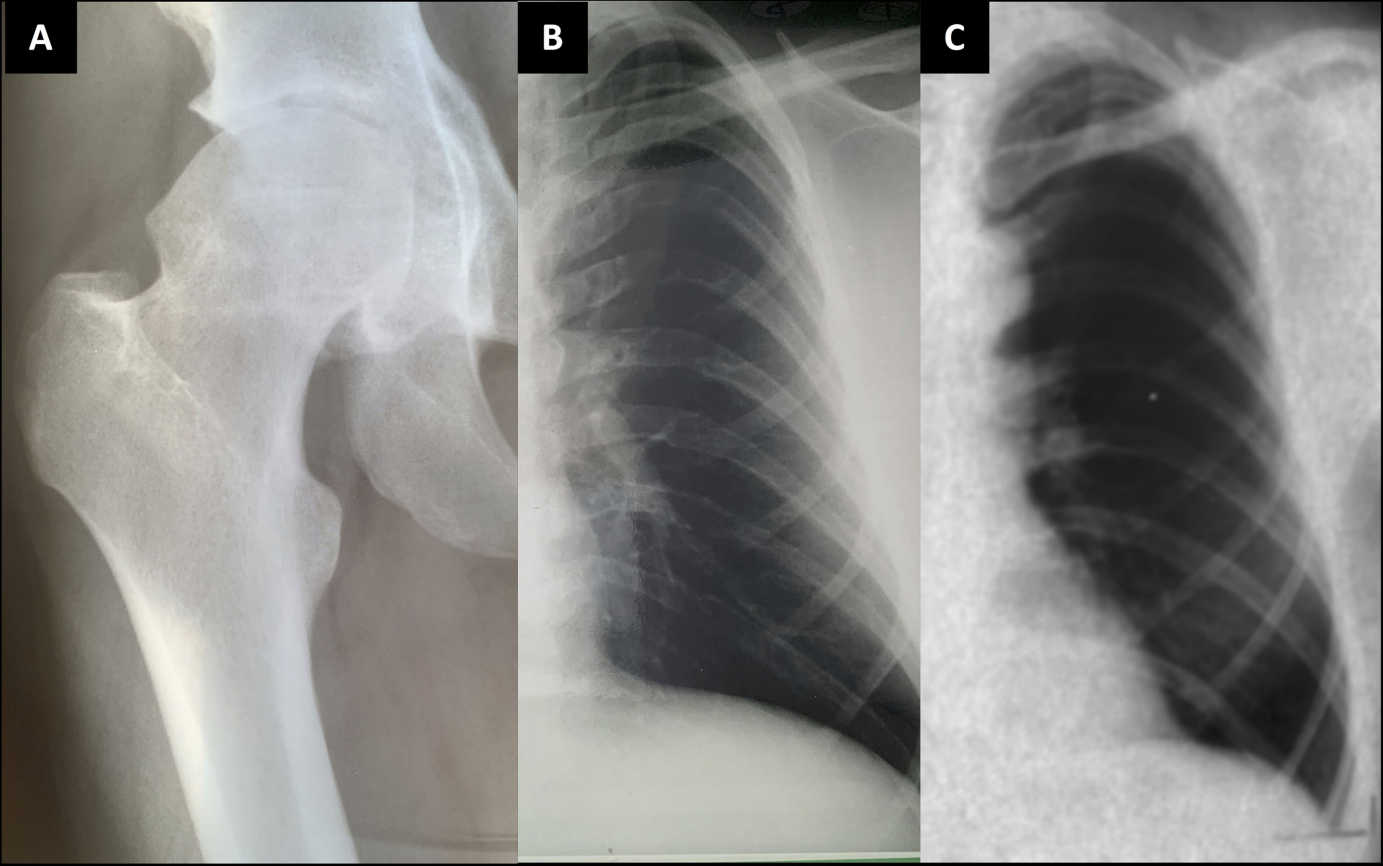
Plain radiographs of the index patient’s father and paternal grandfather. Index patient’s father (II-1) at age 24 **(A, B)**; and the paternal grandfather (I-1) at age 41 **(C)**. Images show slender diaphyses in ribs and degenerative changes on articular surfaces in hip joint with acetabular osteophytes and sclerosis and uneven shape in femoral head.

**Figure 6:**
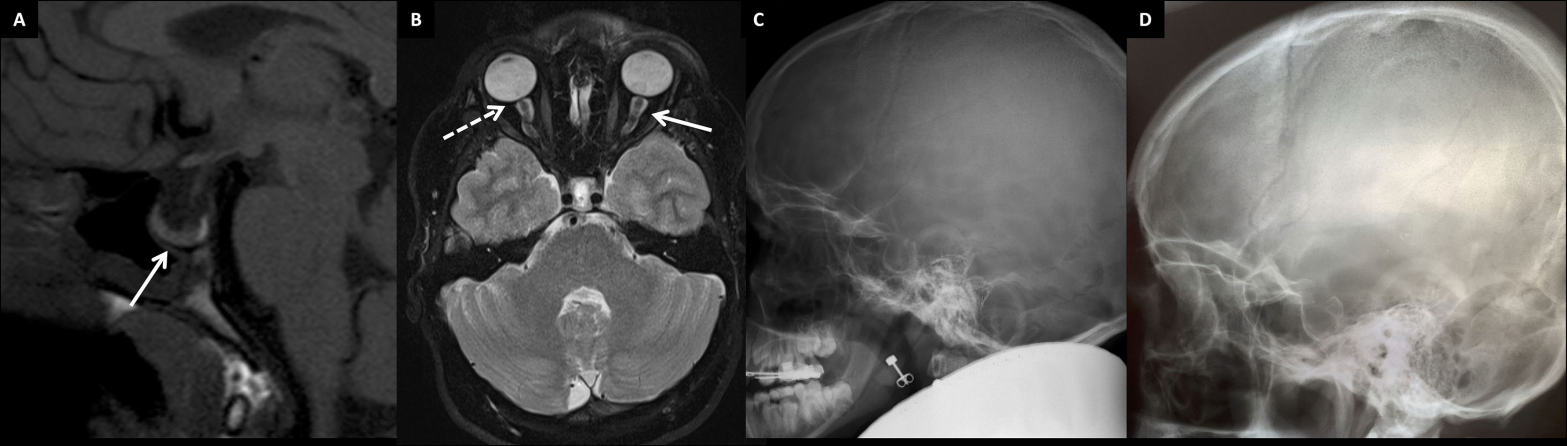
Brain structure findings in two patients with the novel skeletal dysplasia. **(A)** Non-enhanced T1 sagittal MR image showing an enlarged pituitary fossa filled mainly with CSF and pituitary tissue lining the sellar floor (arrow). The bright spot behind the adenohypophysis represents neurohypophysis at the normal position in the 13-year-old female index (III-1). **(B)** Tortuous optic nerves with distended perioptic nerve sheaths (arrow), and optic globe flattening (flattening of the posterior part; dashed arrow) in the 13-year-old female index. Skull radiographs of the female index (III-1) **(C)** and her father (II-1) **(D)** showing an enlarged pituitary fossa in both.

The father’s skeletal radiographs portray similar skeletal changes as in the index: severe arthrosis with joint space narrowing, sclerosis of the subchondral bone plates, cysts and osteophyte formation (**Figures 3**, **5**). Femoral heads appear misshaped and femoral necks short and thick. Spinal radiographs show codfish-shaped vertebrae and balloon-shaped intervertebral discs. His skull radiograph shows an enlarged pituitary fossa (**Figure 6D**), which was later further examined with CT scan of the sella and confirmed to be filled with cerebral fluid consistent with an empty sella.

**The father’s father (I-1)**, deceased at age 44 due to brain hemorrhage, also had a similar phenotype with vertebral anomalies, osteoarthritis and an empty sella. He had suffered prolonged shoulder and knee joint pain requiring cortisone injections, intermittent swelling in smaller joints and one episode of episcleritis. He underwent knee arthrotomy at age 42. He had severe headache episodes and unexplained tremor in his right upper extremity; EEG showed diffuse organic changes. He had no endocrinological abnormalities excluding mild thyrotoxicosis. His spinal radiographs were stated to have shown decreased intervertebral spacing with vertebral codfish-deformities, degenerative osteophytes and cervical–lumbar spondyloarthrosis (images not available). His ribs appeared thin (**Figure 4**) and radiograph of his knee showed chondromalasia. Skull radiographs revealed mild cranial hyperostosis and a large and abnormally shaped sella with a cross-sectional area of 17 x 15 mm. He subsequently underwent pneumoencephalography and was confirmed, by a neurosurgeon and a radiologist, to also have an empty sella.

### Genetic Findings

Screening the WES data for the known genes for OI or skeletal dysplasia identified no pathogenic variants. CNV analysis of known disease genes (>3650), defined as single exon or larger deletions/duplications, similarly revealed no potential variations. Filtering of the WES data for pathogenic single nucleotide variations, small insertions and deletions present only in the index and absent in the unaffected individuals yielded altogether nine candidate variants: four missense variants, three splice region variants and two nonsense variants (**Supplementary Table**). Four of them were neglected for reported associations with different phenotypes to that in our family and other four for unlikely predictions of pathogenicity.

**Figure 7:**
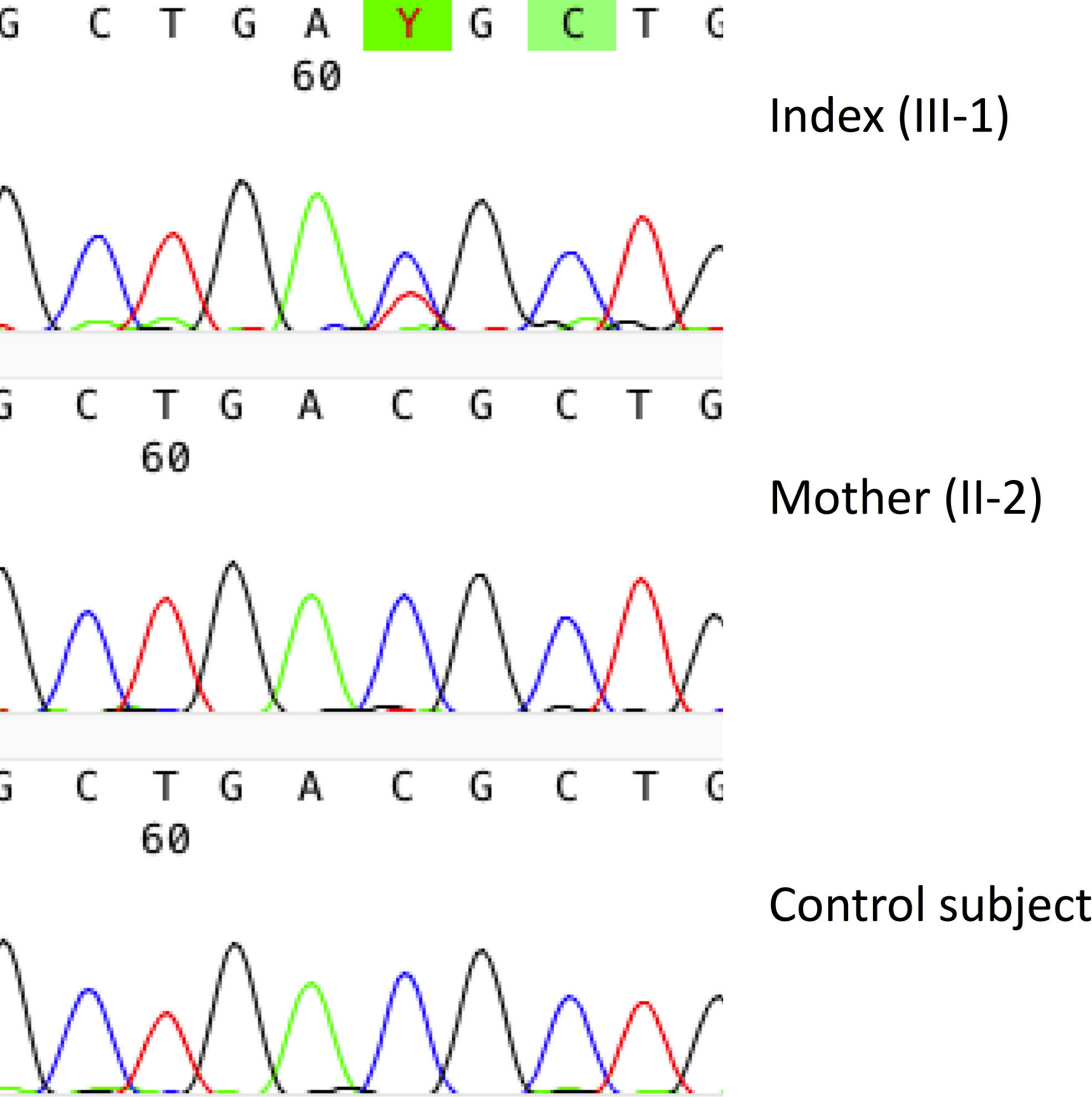
Genetic results in a female patient with a novel *TBX2* missense variant p.Thr300Met. Sanger sequence image of the heterozygous point mutation in the index patient (III-1) and a normal sequence in the healthy mother (II-2).

The identified candidate variant is a novel missense variant c.899C>T (p.Thr300Met) in *TBX2*. The variant is present in 10 individuals in the gnomAD database (MAF 0.00003915 in all the >120,000 exomes and >15,000 genomes), nine of them being Finnish (MAF 0.0003878 in Finnish population alone). All reported carriers are heterozygotes. The variant is predicted “deleterious” and “probably damaging” by SIFT (score 0.01) and PolyPhen2 (score 0.715), respectively. It has a CADD score of 28.9, placing it in the top 1% of all possible reference genome variants. The *TBX2* has a pLoF score of 0.25, indicating low tolerance to inactivation. The Thr residue at 300 is very highly conserved and computed protein modeling by CMBI Hope predicts the mutant protein to be bigger and more hydrophobic than its wildtype version, that the mutation locates in a domain important for the main activity of the protein, and that the mutation might disturb the protein’s function. Furthermore, threonine and methionine have a moderate physicochemical difference, for which reason it is considered a non-conservative substitution (Grantham score = 81) (20). The variant was present only in the index patient and absent in the healthy family members, and its trueness was confirmed by Sanger sequencing (**Figure 7**).

## Discussion

We describe a three-generation Finnish family with early-onset osteochondrodysplasia with vertebral anomalies, severe osteoarthrosis and an empty sella. Using WES, we identified a novel heterozygous missense variant c.899C>T (p.Thr300Met) in *TBX2* that is predicted pathogenic and disease-causing. The variant lies in close proximity to a previously reported disease-causing variant and computed modeling further indicated the location as the protein’s crucial domain with likely damaging functional consequences. Our results support the association between TBX2 and the described skeletal disorder, expanding the clinical symptoms associated with TBX2 and underlining its key role in skeletal and pituitary gland development.

Only few variations in *TBX2*, of both single nucleotides and larger CNVs, have previously been reported in human diseases. To date, missense variants have been identified in altogether four individuals presenting with an overlapping spectrum of craniofacial dysmorphism, congenital cardiac defects, skeletal abnormalities, immune deficiency, endocrine disorders and variable developmental delay (**Table 2**). One of them had a *de novo* missense mutation p.Arg305His—just five amino acids downstream of ours—and consequently growth hormone deficiency, congenital fusions of thoracic spine and hemivertebrae, scoliosis and kyphosis, and triangular facial shape with a high and narrow palate, depressed nasal bridge, epicanthal folds and cupped ears; these were very similar to our index patient (2). The other three individuals harbored a variant p.Arg20Gln and similarly had multi-organ defects, facial dysmorphisms and cleft palate, Klippel–Feil vertebral anomalies and congenital absence of the thymus (2). Functional analyses of both variants in HEK293 cells revealed the variants to reduce the protein’s transcriptional activity. In addition to these, two papers have reported syndromes following larger genetic defects involving *TBX2*. Radio et al. identified a duplication of 17q23.2 chromosome region, encompassing *TBX2*, in a 4-year-old male patient with mild skeletal anomalies with phalangeal hypoplasia, growth retardation, brain tissue defects and facial dysmorphia, and Nimmakayalu et al. reported a female patient with a microdeletion encompassing both *TBX2* and *TBX4* and consequent musculoskeletal and limb anomalies, microcephaly, hypotelorism and moderate developmental delay (4, 21). Lastly, a variant rs882367 in *TBX2* has genome-wide significant association with sitting height ratio—an abnormal finding in our patients (5).

**Table 2:** Clinical characteristics in the three Finnish patients with a novel skeletal dysplasia, compared with previously reported findings in *TBX2* mutation-positive individuals.

Clinical finding	Patients with missense variant p.R20Q*	Patient with missense variant p.R305H*	Our patients with missense variant p.T300M
Growth	Height 3^rd^ to 36^th^ percentile	Height 2^nd^ percentile	Normal height
Facial dysmorphism	Triangular face, hypertelorism with epicanthal folds, depressed nasal bridge, cupped and low set ears	Triangular face, depressed and broad nasal tip, cupped and low set ears	Hypertelorism, epicanthal folds, depressed nasal bridge with broad bulbous tip, low set, slightly cupped ears
Orofacial clefting	Cleft palate	High arched, narrow palate	High arched, narrow palate
Skeletal	Klippel-Feil anomaly, rib fusions, scoliosis, camptodactyly, Sprengel deformities	Fusions of thoracic spine, scoliosis and kyphosis	Mild kyphosis and scoliosis, codfish-type vertebral dysplasia, thin ribs and long-bone diaphyses
Neurology, intellectual ability	Normal to autistic behaviors, neonatal seizures	Mild cognitive impairments	Empty sella, headaches, depression
Endocrine	Autoimmune thyroiditis, hypoparathyroidism	Growth hormone deficiency	Mild thyrotoxicosis and parathyroidism, otherwise normal endocrinology
Arthrosis	None reported	None reported	Early-onset osteoarthritis, iritis/episcleritis, biochemistry for rheumatoid arthritis normal
Cardiology	Atrial septal defect, patent ductus arteriosus	Double outlet right ventricle, valvular pulmonic stenosis	High blood pressure, no cardiac abnormalities

*As reported by 2.

*TBX2* encodes a transcriptional activator/repressor with important roles in skeletal development, specifically in limbs and craniofacial structures. In the developing bone, TBX2 is expressed especially highly in hypertrophic chondrocytes as well as in osteogenic progenitors and osteoblasts (22, 23). It also shows regulatory effects on the expression of *connexin 34* and *collagen type 1*—both crucial for normal ossification and osteoblast function and associated with craniofacial abnormalities and connective tissue disorders, respectively (23, 24). *In vivo* experiments have also shown that Tbx2 is expressed in the developing palate and other facial structures and that *Tbx2*-deficient mice exhibit premature pause and incomplete fusion of the opposing palatal shelves (11). Furthermore, following homozygous *Tbx2* knockout, mice also portray polydactyly (13, 25, 26). Taken together, although no digit anomalies were observed, the vertebral abnormalities, facial dysmorphism and arthritis in our patients resonate with the above-described functions of TBX2.

Alongside the skeletal changes, the finding of an empty sella in all three individuals is an interesting and a novel finding. Primary empty sella is usually an incidental finding with an unknown etiology. Especially in children, it has been reported in several genetic disorders, such as Turner syndrome, Moyamoya disease, Bartter’s syndrome and Prader–Labhart–Willi syndrome (27). Although its pathogenesis has been linked to incomplete development of the sellar diaphragm in coexistence with suprasellar factors inflicting an increased pressure (27), the exact molecular mechanisms are still unknown. TBX2 has been implicated in development of the hypothalamus–pituitary-axis and although this has been demonstrated in a few *in vivo* studies, the exact molecular mechanisms remain unclear (28–30). Further, empty sella is also considered a radiologic sign of intracranial hypertension, in which the pituitary tissue is protruded and flattened against the sellar floor (31). This, together with the finding of changes in the optic nerve in the index patient could also suggest that the empty sella may be secondary to elevated intracranial pressure. Although none of the previously described *TBX2* mutation-positive patients had an empty sella—none of them were evaluated for it, however—the patient with the mutation p.Arg305His had growth hormone deficiency (2). Furthermore, although neither patient had ophthalmic problems or evident endocrine abnormalities as often associated with empty sella syndrome, the patients had severe headaches, obesity and several psychiatric symptoms, which are likely related to their genetic condition and the presumed abnormal hypothalamus–pituitary function of TBX2. Lastly, skeletal dysplasia with pituitary abnormalities is rare and only a few have been described, such as e.g., pycnodysostosis with osteosclerosis, severe skeletal abnormalities and hypopituitarism (32); Cantú syndrome with skeletal and cardiac anomalies and hypertrichosis (33); and patients with *SEMA3A* variants and a complex phenotype including skeletal and pituitary dysplasia (34).

We acknowledge our study to be limited in certain aspects. The lack of DNA samples from the father and grandfather prevented us from fully testing the variant’s segregation with the phenotype. Similarly, we were unable to evaluate the variant’s significance on the protein’s function in *in vitro* or *in vivo* settings in the scope of this study. Furthermore, evaluation of the father and grandfather were further limited by the lack of complete clinical data and radiologic images. The full phenotypic spectrum and functional relevance of the identified variant therefore warrant further investigation.

In conclusion, we report a novel skeletal dysplasia with rheumatoid arthritis-like joint degeneration and an empty sella associating with a pathogenic *TBX2* variant. Although our clinical findings share close similarities with previously reported characteristics in subjects with *TBX2* variants, our patients also exhibit codfish vertebrae and empty sella, both previously undescribed in *TBX2* mutation-positive patients. Our results resonate with known roles of TBX2 in skeletal and central nervous system development and also underpin the complexity and still partly unknown genetic landscape of skeletal dysplasias. Future reports on other *TBX2* variants in skeletal disorders will be helpful in further delineating the TBX2-associated skeletal dysplasia.

## Data Availability Statement

The datasets presented in this article are not readily available because the hospital ethics guidelines prevent distribution of individualized genomic data. Requests to access the datasets should be directed to riikka.makitie@helsinki.fi.

## Ethics Statement

The studies involving human participants were reviewed and approved by Institutional Ethics Board at Children’s Hospital, Helsinki University Hospital, Finland. The patients/participants provided their written informed consent to participate in this study.

## Author Contributions

Study design: RM and OM. Study conduct: all authors. Data collection: RM and IK. Data analysis: RM, ST-S, and IK. Drafting manuscript: RM. Revising manuscript content: all authors. RM and OM take responsibility for the integrity of the data. All authors contributed to the article and approved the submitted version.

## Funding

This study was supported by the Academy of Finland (277843), the Sigrid Jusélius Foundation, the Folkhälsan Research Foundation, the Novo Nordisk Foundation (21322), the Foundation for Pediatric Research, Konung Gustaf V:s och Drottning Victorias Frimurarestiftelse, the Swedish Research Council and the Stockholm County Council, the Orion Research Foundation, the Finnish ORL–HNS Foundation, and the Juhani Aho Foundation.

## Conflict of Interest

The authors declare that the research was conducted in the absence of any commercial or financial relationships that could be construed as a potential conflict of interest.

## Publisher’s Note

All claims expressed in this article are solely those of the authors and do not necessarily represent those of their affiliated organizations, or those of the publisher, the editors and the reviewers. Any product that may be evaluated in this article, or claim that may be made by its manufacturer, is not guaranteed or endorsed by the publisher.
