# Supplementary material for: A Novel Osteochondrodysplasia With Empty Sella Associates With a TBX2 Variant
Source: Front Endocrinol (Lausanne). 2022 Mar 3;13:845889. doi: 10.3389/fendo.2022.845889 (PMC8927981; doi:10.3389/fendo.2022.845889)
Supplement: Supplementary file 1 [file Table_1.docx]

**A novel osteochondrodysplasia with empty sella**

**associates with a *TBX2* variant**

**Supplementary Table.** Other potential heterozygous variants in the Finnish family with autosomal dominant osteochondrodysplasia and empty sella.

| **Gene** | **Variant (hg19)** | **Variant type** | **MAF gnomAD* (%)** | **CADD** | **SIFT** | **Polyphen 2** | **Mutation Taster2** | **Syndromes linked** | **Reason for exclusion** |
| --- | --- | --- | --- | --- | --- | --- | --- | --- | --- |
| *TBX2* | ENST00000240328:  c.899C>T,  p.Thr300Met | Missense | 4.10E-05 | 28.3 | Deleterious | Possibly damaging | Disease causing |  |  |
| *PTGR1* | ENST00000238248.3:  c.-217–6_-217–4del,  g.6868_6870delTTG | Splice region | Novel | None | - | - | Disease causing | - | Associated with prostaglandin metabolism |
| *ABCF3* | ENST00000292808.5:  c.1510C>A,  p.Arg504Ser | Missense | 1.62E-05 | 17.46 | Tolerated | Benign | Polymorphism | ABCD syndrome,  CACH syndrome | Dissimilar phenotype/non-pathogenic |
| *EOMES* | ENST00000295743.4:  c.747_759del, p.Gly250AspfsTer34 | Frameshift | Novel | None | - | - | Disease causing | Microcephaly-polymicrogyria-corpus callosum agenesis syndrome, bilateral polymicrogyria | Dissimilar phenotype |
| *KRT10* | ENST00000269576.5:  c.1684_1685insAGC...AGC,  p.Ser562Ter | Stop-gained | Novel | None | - | - | Disease causing | Annular epidermolytic ichthyosis | Dissimilar phenotype |
| *FAM216B* | ENST00000313851.1:  c.99+6_99+7insTT,  g.2623_2624insTT | Splice region | Novel | None | - | - | Polymorphism | - | Non-pathogenic |
| *KIAA1551* | ENST00000312561:  c.449C>T,  p.Pro150Leu | Missense | 4.88E-05 | 7.9 | Tolerated | Benign | Polymorphism | - | Non-pathogenic |
| *MGP* | ENST00000228938:  c.55A>G,  p.Cys19Arg | Missense | Novel | 24.1 | Deleterious | Probably damaging | Disease causing | Keutel syndrome | Dissimilar phenotype |
| *KRR1* | ENST00000229214:  c.394–7_394–6insT,  g.5021_5022insT | Splice region | Novel | None | - | - | Polymorphism | Duodenum adenoma, hereditary nonpolyposis colorectal cancer | Dissimilar phenotype/non-pathogenic |

* all populations

MAF, minor allele frequency

hg19, reference genome
